# Comparative economic evaluation of quetiapine plus lamotrigine combination vs quetiapine monotherapy (and folic acid vs placebo) in patients with bipolar depression (CEQUEL)

**DOI:** 10.1111/bdi.12713

**Published:** 2018-12-17

**Authors:** Judit Simon, John R. Geddes, Alexandra Gardiner, Jennifer Rendell, Guy M. Goodwin, Susanne Mayer

**Affiliations:** ^1^ Department of Health Economics, Center for Public Health Medical University of Vienna Vienna Austria; ^2^ Department of Psychiatry University of Oxford and Oxford Health NHS Trust, Warneford Hospital Oxford UK; ^3^ Health Economics Research Centre, Nuffield Department of Population Health University of Oxford Oxford UK; ^4^ Nuffield Department of Primary Care Health Sciences University of Oxford Oxford UK

**Keywords:** bipolar disorder, cost, cost‐effectiveness, cost‐utility, depression, economic evaluation, quality of life

## Abstract

**Objectives:**

Although not licensed for acute bipolar depression, lamotrigine has evidence for efficacy in trials and its use is recommended in guidelines. So far there had been no prospective health economic evaluation of its use.

**Methods:**

Cost‐utility analysis of the CEQUEL trial comparing quetiapine plus lamotrigine vs quetiapine monotherapy (and folic acid vs placebo in an add‐on factorial design) for patients with bipolar depression (n = 201) from the health and social care perspective. Differences in costs together with quality‐adjusted life years (QALYs) between the groups were assessed over 52 weeks using a regression‐based approach.

**Results:**

Health‐related quality of life improved substantially for all randomization groups during follow‐up with no significant difference in QALYs between any of the comparisons (mean adjusted QALY difference: lamotrigine vs placebo −0.001 (95% CI: −0.05 to 0.05), folic acid vs placebo 0.002 (95% CI: −0.05 to 0.05)). While medication costs in the lamotrigine group were higher than in the placebo group (£647, *P* < 0.001), mental health community/outpatient costs were significantly lower (−£670, *P* < 0.001). Mean total costs were similar in the groups (−£180, *P* = 0.913).

**Conclusions:**

Lamotrigine improved clinical ratings in bipolar depression compared with placebo. This differential effect was not detected using the EQ‐5D‐3L. The additional cost of lamotrigine was balanced by significant savings in some other medical costs which made its use cost neutral to the health service. Compared to placebo, folic acid produced neither clinical nor significant health economic benefits. The study supports the use of lamotrigine in combination with other drugs to treat bipolar depression.

## 1 INTRODUCTION

Worldwide, mental and substance abuse disorders accounted for 8.6 million years lived with disability (YLD) in 2010, making it the number one cause of YLDs. 7.4% of it are attributed to bipolar disorders,^1^ ranking them 18th for all global years lived with disability and 6th of all mental and behavioural disorders.^2^ Bipolar disorders are associated with reduced life expectancy by 11‐20 years^3^ and a 20% higher risk of suicide^4^ compared to the general population as well as reduced quality of life for both the people affected^5^ and their families.^6^ These adverse outcomes are especially related to the depressive episodes of bipolar disorders. In addition, bipolar disorders cause high direct healthcare costs and substantial societal costs due to patients’ production losses.^7^

Quetiapine or lamotrigine is among the few treatment options recommended by NICE (National Institute for Health and Care Excellence) guidelines^8^ for treatment of bipolar depression. However, monotherapy is associated with limited efficacy or, in the case of lamotrigine, switch to mania, while combination therapies appear to be associated with better outcomes.^9^ Thus, lamotrigine in combination with lithium therapy was found to be effective in the LamLit trial.^10^ The CEQUEL trial investigated if quetiapine combined with lamotrigine resulted in greater improvement in depressive symptoms than quetiapine monotherapy. Since there was also some evidence that folic acid, a widely accessible over‐the‐counter drug, is an effective treatment option for patients with unipolar depression,^11^ its add‐on effect was also investigated using a factorial design.

In the CEQUEL trial, a lower mean QIDS‐SR16 (Quick Inventory of Depressive Symptomatology—self report version) total score for the group receiving lamotrigine vs the placebo group was observed at 12 weeks (−1.73, 95% CI: −3.57 to 0.11, *P* = 0.066) and at 52 weeks (−2.69, 95% CI: −4.89 to −0.49, *P* = 0.017), implying that quetiapine‐lamotrigine combination therapy improved depressive symptoms more than quetiapine alone. Accordingly, lamotrigine's use was recommended in the recent BAP (British Association for Psychopharmacology) bipolar guidelines.^12^ Addition of folic acid was not found to be superior to placebo but, rather, to reduce the effect of lamotrigine.^9^

In fact, the adoption of lamotrigine has lagged behind that of other new drugs used in bipolar disorders in the UK.^13^ This probably reflects the absence of a marketing authorization for its use in bipolar depression. Therefore, evidence for efficacy and cost‐effectiveness from independent clinical trials is of critical importance in improving practice. The analysis of further data from CEQUEL on quality of life, costs and cost‐effectiveness over 52 weeks of treatment of patients with bipolar disorders here contributes a complementary perspective for considering the regular use of lamotrigine.

## 2 MATERIALS AND METHODS

### 2.1 Study design and study population

**Figure 1:**
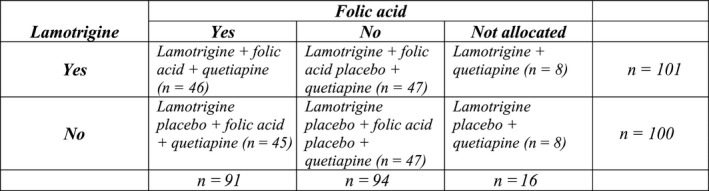
Group randomization

CEQUEL was a multi‐centre (27 sites), double‐blind, randomized, placebo‐controlled, parallel group, 2 × 2 factorial clinical trial conducted in the UK (for more details on the clinical study, see Geddes et al^9^). The clinical trial was registered with EudraCT, number 2007‐004513‐33 and approved by the Oxfordshire REC B ethics committee. Patient inclusion criteria were a primary diagnosis of bipolar disorder type I or II based on DSM‐IV^14^ (Diagnostic and Statistical Manual of Mental Disorders—IV) criteria for hypomanic or manic episode, a current depressive episode requiring new pharmacological treatment, informed consent and aged 16 years or over. Following a 7‐14 days run‐in phase on quetiapine monotherapy, 202 participants were randomized to the following added medication (see Figure 1 for an overview): 101 participants to lamotrigine (200 mg/d; 100 mg/d with concurrent valproate and 400 mg/d with concurrent combined oral contraceptives), 101 to placebo lamotrigine. Additionally, participants not currently taking folic acid and without contraindications to do so were assigned to folic acid (500 µg/d) or placebo folic acid. Out of the 202 study participants, 94 participants were thus separately randomized to folic acid and 92 participants to placebo folic acid. Sixteen participants were not randomized to the folic acid/placebo comparison. One participant died during the 52 weeks follow‐up (suicide; group allocation: placebo lamotrigine/active folic acid) and was excluded from the health economic analysis. Table 1 presents the baseline characteristics of the 201 analysed participants.

**Table 1:** Patient characteristics at baseline (n = 201)

	Placebo (n = 100)		Lamotrigine (n = 101)		Placebo (n = 94)		Folic acid (n = 91)		NA^a^ (n = 16)	
	N	% or mean (SD)	N	% or mean (SD)	N	% or mean (SD)	N	% or mean (SD)	N	% or mean (SD)
Age (y)										
≤30	17	17%	19	19%	19	20%	17	19%	0	0%
31‐40	20	20%	25	25%	25	27%	17	19%	3	19%
41‐50	31	31%	34	34%	26	28%	32	35%	7	44%
>50	32	32%	23	23%	24	26%	25	28%	6	38%
Sex										
Male	45	45%	44	44%	41	44%	41	45%	7	44%
Female	55	55%	57	56%	53	56%	50	55%	9	56%
Bipolar type										
I	75	75%	74	74%	67	71%	69	76%	13	81%
II	25	25%	27	27%	27	29%	22	24%	3	19%
Dose of quetiapine (mg/d)										
≤150	19	19%	19	19%	16	17%	21	23%	1	6%
>150‐<300	14	14%	18	18%	20	21%	9	10%	3	19%
300	53	53%	55	54%	50	53%	49	54%	9	56%
>300	14	14%	9	9%	8	9%	12	13%	3	19%
Concurrent medication										
Lithium	14	14%	12	12%	13	14%	12	13%	1	6%
Valproate	18	18%	24	24%	22	23%	18	20%	2	13%
Other mood stabilizer	2	2%	5	5%	2	2%	3	3%	2	13%
Olanzapine	4	4%	3	3%	5	5%	1	1%	1	6%
Other atypical antipsychotic	3	3%	4	4%	4	4%	3	3%	0	0%
Conventional antipsychotic	3	3%	2	2%	1	1%	4	4%	0	0%
Antidepressant	39	39%	29	29%	29	31%	32	35%	7	44%
Pre‐trial quetiapine	22	22%	25	25%	23	24%	21	23%	3	19%
Pre‐trial lamotrigine	1	1%	1	1%	1	1%	1	1%	0	0%
Participants with mood episodes in past year	26	26%	26	26%	27	29%	23	25%	2	13%
Accommodation										
In own home (rented or owned)	73	73%	70	69%	62	66%	68	75%	13	81%
In a relative's or friend's home	9	9%	12	12%	13	14%	7	8%	1	6%
In sheltered accommodation	1	1%	3	3%	1	1%	3	3%	0	0%
Other	2	2%	1	1%	2	2%	0	0%	1	6%
Missing	15	15%	15	15%	16	17%	13	14%	1	6%
Employment										
Full‐time	15	15%	21	21%	18	19%	16	18%	2	13%
Part‐time	14	14%	15	15%	16	17%	12	13%	1	6%
Not in paid employment	63	63%	61	60%	55	59%	56	62%	13	81%
Missing	8	8%	4	4%	5	5%	7	8%	0	0%
EQ‐5D	98	0.500 (0.309)	99	0.516 (0.300)	92	0.505 (0.298)	90	0.525 (0.309)	15	0.427 (0.315)
Missing	2		2		2		1		1	

Note

Excluding the participant who died during the trial. Percentages may not add up to 100% due to rounding. Participants were randomized to lamotrigine or placebo. In addition, participants were randomized to folic acid or placebo if consenting to that part of the study. Participants are therefore counted twice in this table.

N, number of participants; NA, not allocated; SD, standard deviation.

a Participants who opted not to be randomized into the folic acid part of the study.

### 2.2 Data collection

Health‐related quality of life information was provided by the participants using the TrueColours system via text message, email or paper at baseline, 12 weeks and 52 weeks. Resource use data were collected via self‐administered questionnaires and complemented with clinical records where possible. Data collection took place between October 2008 and April 2013. The collection of resource use data was based on an amended version of the Client Service Receipt Inventory (CSRI) instrument,^15^ a widely used and validated resource use measurement instrument in mental health. Collected resource use information included all hospital and community health and social service use (categorized as mental health community/outpatient, mental health inpatient, non‐mental health outpatient (eg, utilization of alternative therapists, non‐psychiatric outpatient clinics, day patient admissions to a medical/surgical wards, accident and emergency visits and other), non‐mental health inpatient, primary care, social care), psychiatric medication (trial and non‐trial) and lost productivity (absenteeism) (Table 2).

**Table 2:** Key unit costs used to value resource use (measured in £, 2013/14 prices)

Resource use	Unit costs (£)	Unit	Source of estimate
Psychiatric medication			
Oral medication	various	per daily dose (mg)	BNF 67 (March—September 2014)^16^
Mental health inpatient			
Psychiatric hospital inpatient general ward	348.9	per day	Curtis (2012)^a^^,^ 17
Psychiatric hospital inpatient PICU (Psychiatric Intensive Care Unit)	675.1	per day	Curtis (2012)^a^^,^ 17
Non‐mental health inpatient			
Other medical/surgical inpatient department	466.3‐1241.2	per day	Scottish National Tariff 2013‐2014^18^
Mental health community/outpatient			
Psychiatrist—phone contact	28.4	per contact	NHS reference costs 2013‐14,^19^ Curtis (2014)^20^
Psychiatrist—contact at health or social service	47.0	per visit	NHS reference costs 2013‐14,^19^ Curtis (2014)^20^
Psychiatrist—contact at home or in community	117.5	per visit	NHS reference costs 2013‐14,^19^ Curtis (2014)^20^
Psychologist—phone contact	16.3	per contact	Curtis (2014)^20^
Psychologist—contact at health or social service	138.0	per visit	Curtis (2014)^20^
Psychologist—contact at home or in community	173.9	per visit	Curtis (2014)^20^
Community Mental Health Nurse (CPN)—phone contact	6.6	per contact	Curtis (2014)^20^
Community Mental Health Nurse (CPN)—contact at health or social service	16.5	per visit	Curtis (2014)^20^
Community Mental Health Nurse (CPN)—contact at home or in community	34.7	per visit	Curtis (2014)^20^
Drug/alcohol service worker—phone contact	29.1	per contact	Curtis (2014)^20^
Drug/alcohol service worker—contact at health or social service	48.0	per visit	Curtis (2014)^20^
Drug/alcohol service worker—contact at home or in community	120.0	per visit	Curtis (2014)^20^
Attendance of day centre (ie, groups/programmes not run by health staff)	38.2	per session	Curtis (2012)^a^^,^ 17
Drop‐in centre (including street agencies)	38.2	per session	Curtis (2012)^a^^,^ 17
Self‐help/support group	60.9	per session	Curtis (2012)^a^^,^ 17
Non‐mental health outpatient			
Alternative therapies (NHS)—visits using NHS services	43.8	per visit	NHS Choices (2014)^21^
Alternative therapies (NHS)—visits using private services	69.2	per visit	Private Healthcare Tariff (2012)^a^^,^ 22
Day patient hospital attendance—medical ward	206.3	per attendance	Scottish National Tariff 2013‐2014^18^
Day patient hospital attendance—surgical ward	233.2	per attendance	Scottish National Tariff 2013‐2014^18^
Day patient hospital attendance/Accident and emergency visit	135.1	per visit	NHS reference costs 2013‐14^19^
Other medical/surgical outpatient visits	3.0‐3294.6	per visit	NHS reference costs 2013‐14,^19^ Curtis (2012)^a^^,^,^17^ Curtis (2014)^20^
Primary care			
GP—phone contact	23.0	per contact	Curtis (2014)^20^
GP—contact at health or social service	38.0	per visit	Curtis (2014)^20^
GP—contact at home or in community	96.1	per visit	Curtis (2014)^20^
Practice nurse (at GP clinic)—phone contact	4.4	per contact	Curtis (2014)^20^
Practice nurse (at GP clinic)—contact at health or social service	11.0	per visit	Curtis (2014)^20^
Practice nurse (at GP clinic)—contact at home or in community	23.1	per visit	Curtis (2014)^20^
Social care			
Social worker—phone contact	9.2	per contact	Curtis (2014)^20^
Social worker—contact at health or social service	18.3	per visit	Curtis (2014)^20^
Social worker—contact at home or in community	23.1	per visit	Curtis (2014)^20^
Community support worker (unqualified)—phone contact	3.3	per contact	Curtis (2014)^20^
Community support worker (unqualified)—contact at health or social service	6.7	per visit	Curtis (2014)^20^
Community support worker (unqualified)—contact at home or in community	8.4	per visit	Curtis (2014)^20^
Home help/home care worker—phone contact	6.2	per contact	Curtis (2014)^20^
Home help/home care worker—contact at health or social service	27.8	per visit	Curtis (2014)^20^
Home help/home care worker—contact at home or in community	27.8	per visit	Curtis (2014)^20^
Housing worker—phone contact	2.6	per contact	Assuming national average salary, ONS (2014)^23^
Housing worker—contact at health or social service	5.3	per visit	Assuming national average salary, ONS (2014)^23^
Housing worker—contact at home or in community	6.7	per visit	Assuming national average salary, ONS (2014)^23^
Voluntary/Charity worker—phone contact	2.6	per contact	Assuming national average salary, ONS (2014)^23^
Voluntary/Charity worker—contact at health or social service	5.3	per visit	Assuming national average salary, ONS (2014)^23^
Voluntary/Charity worker—contact at home or in community	6.7	per visit	Assuming national average salary, ONS (2014)^23^
Indirect costs			
Lost productivity (sick leave)	103.6	per day	Assuming national average salary, ONS (2014)^23^

a Adjusted for inflation based on the hospital and community health services (HCHS) pay and prices index.^20^

### 2.3 Outcomes

The primary economic analysis was an incremental cost‐utility analysis. Outcomes were expressed as QALYs and calculated as the area under the curve based on the EQ‐5D‐3L data. EQ‐5D‐3L is a standardized, non‐disease‐specific instrument designed for valuing general health‐related quality of life.^24^ It is a generic, self‐reported measure of health‐related quality of life alongside five dimensions (mobility, self‐care, usual activities, pain/discomfort and anxiety/depression) which allows the preference‐based, comparative evaluation of distinctively different treatment options and disease areas and is therefore the preferred outcome measure to be used in economic evaluations by NICE.^25^ Although EQ‐5D was found sensitive to changes in health‐related quality of life of bipolar patients during their depressive episodes, its sensitivity to change in episodes of mania or mixed episodes however remains unclear.^26^ EQ‐5D utilities were based on the UK tariff values.^27^

### 2.4 Costs

UK national‐level unit costs were applied for each resource use item to calculate total costs of resources used (Table 2). All unit costs referred to the financial year 2013/14 to match the last year of measured resource use and were expressed in British Pounds (£). Medication costs were calculated on the basis of daily dose information, which was multiplied with the average (proprietary and non‐proprietary) unit price per milligram for each compound taken from the British National Formulary.^16^ Lost productivity was measured based on the human capital approach. For participants in employment, absent work days were multiplied by the average daily UK national salary.^23^

### 2.5 Analyses

In line with NICE guidelines, cost‐effectiveness was primarily assessed from the health and social care perspective.^25^ To account for the high indirect costs associated with bipolar disorders,^28^ indirect costs (lost productivity costs) were also measured (Supporting Information Table S2).

To accommodate for the factorial design of the trial and to be in line with the main clinical analysis, a regression‐based approach using ordinary least squares (OLS) was adopted to analyse mean outcomes and mean costs by randomization group. This technique not only allows adjustment for the effect of the second intervention (ie, folic acid in the analysis on lamotrigine and lamotrigine in the analysis on folic acid), but also to control for covariates and to deal with different sample sizes between groups.^29^ As some patients chose not to be randomized into the folic acid component (n = 16), two dummy variables indicating whether or not lamotrigine was given (A; placebo: n = 100, lamotrigine: n = 101) and whether or not folic acid was given (B; placebo: n = 94, folic acid: n = 91, opted out of the folic acid component: n = 16) were included in the regression in addition to the covariates age and sex and baseline differences in the relevant measure, capturing the different sub‐groups of patients. In the main analysis, no interaction effect was assumed. In the analysis of the folic acid comparison, only the participants randomized to the folic acid component of the study were included, therefore excluding the patients (n = 16) that were not randomized to the folic acid/placebo comparison.

All analyses were done on an intention‐to‐treat basis. A p value less than 5% was considered as statistically significant. Microsoft^®^ Excel 2013 was used for costing and Stata^®^ 13.1 for the statistical analyses.

#### 2.5.1 Outcomes

EQ‐5D utility analyses were based on two main approaches: available cases analysis and full dataset analysis following multiple imputation of missing data. For the multiple imputation, we used covariates such as the previous EQ‐5D‐3L utility values in addition to other covariates matching the main clinical analysis such as group allocation, number of lifetime episodes of depression and mania, type of bipolar disorder (I or II), age, sex, dose of quetiapine (<300 mg/d; 300+ mg/d), concurrent medication, pre‐screening treatment and number of mood episodes in the past year (<4 or >=4).^8^, ^30^ The number of imputation sets was set to match the percentage of incomplete cases.^31^ A regression‐based approach adjusting for randomization group, baseline EQ‐5D utility, age and sex was applied to estimate mean EQ‐5D utility values and QALYs (Table 3).^32^ In the main analysis, transitions in health‐related quality of life between 0 and 12 weeks as well as 12 and 52 weeks were assumed to be linear. Further sensitivity analyses were carried out assuming the health state transitions to occur at the beginning and at the end of each period, respectively.

**Table 3:** EQ‐5D‐3L utility and QALY mean differences between groups for available cases and the full imputed dataset, regression adjusted

	N	Adjusted mean difference	95% LCL	95% UCL	*P* value
Lamotrigine vs placebo					
Available cases					
EQ‐5D: 12 wk	152	0.009	−0.08	0.10	0.848
EQ‐5D: 52 wk	92	0.013	−0.09	0.12	0.807
Imputed full dataset					
EQ‐5D: 12 wk	201	0.007	−0.06	0.08	0.842
EQ‐5D: 52 wk	201	−0.012	−0.06	0.04	0.656
QALY: 52 wk	201	−0.001	−0.05	0.05	0.972
Folic acid vs placebo^a^					
Available cases					
EQ‐5D: 12 wk	140	−0.006	−0.10	0.09	0.905
EQ‐5D: 52 wk	84	0.095	−0.01	0.20	0.085
Imputed full dataset					
EQ‐5D: 12 wk	185	−0.031	−0.11	0.04	0.421
EQ‐5D: 52 wk	185	0.046	−0.01	0.10	0.098
QALY: 52 wk	185	0.002	−0.05	0.05	0.938

Notes

LCL, lower confidence level; N, number of participants; UCL, upper confidence level.

a Folic acid comparison restricted to those participants who consented to separate randomization.

#### 2.5.2 Costs

For inpatient resource use and medication, data for the full sample (n = 201) were available from the questionnaire complemented with the clinical records. For other health and social care resource use information, there were high rates of missing data mostly due to loss to follow‐up and withdrawal. Only 119 (59%) patients had relevant information with 71 (35%) patients having complete information. Missing data were handled in multiple steps. Firstly, missing health and social care costs as well as lost productivity was calculated using extrapolation for those patients with partial health and social care data to account for missing data due to loss to follow‐up and withdrawal (n = 38). Secondly, health and social care costs were calculated following multiple imputation of missing data for the 82 patients with no cost information. Multiple imputation was based on group allocation, age, sex, EQ‐5D utility values, other costs for the same time period and costs from the previous period if relevant. The number of imputation sets was set to match the percentage of missing cases. Lost productivity was calculated for all participants who reported employment at some point during the clinical trial (n = 47).

Three sensitivity analyses were conducted. Firstly, the effect of excluding three outlier patients due to extremely high inpatient costs (Supporting Information Table S1) was explored (n = 198). Secondly, the cost analysis was restricted to available cases only to assess the impact of missing values (Supporting Information Table S2) (n = 119). Finally, the potential interaction effects between randomization groups were investigated for all participants randomized to the lamotrigine and folic acid comparison (Supporting Information Table S3) (n = 185).

#### 2.5.3 Cost‐effectiveness

The main analyses of QALYs (Table 3) and total health and social care costs (Table 4) were based on the regression adjusted, full imputed dataset over 52 weeks. Results are presented as differences in mean outcomes and costs between the randomized groups at 52 weeks. No discounting was necessary either for costs or for outcomes. To generate a joint distribution of the mean incremental costs and mean incremental effects and illustrate uncertainty, non‐parametric bootstrapping was carried out for both group comparisons.^33^

**Table 4:** Imputed, regression adjusted annual mean costs per participant (in £ for the year 2013/14)

	Placebo			Lamotrigine			Lamotrigine vs. placebo			
	Mean	SE	N	Mean	SE	N	Mean difference	95% LCL	95% UCL	*P* value
Total health and social care costs	6003.18	1162.09	100	5823.28	1156.31	101	−179.90	−3416.84	3057.04	0.913
Total medication costs	798.94	77.12	100	1512.33	76.74	101	713.39	498.57	928.20	<0.001
Trial medication	670.55	68.40	100	1317.53	68.06	101	646.98	456.45	837.52	<0.001
Other medication	128.39	34.90	100	194.80	34.73	101	66.40	−30.82	163.62	0.180
Total hospital costs	2863.48	1113.15	100	2432.67	1107.61	101	−430.81	−3531.41	2669.80	0.784
Mental health inpatient	2773.01	1063.19	100	1916.39	1057.90	101	−856.62	−3818.07	2104.84	0.569
Non‐mental health inpatient	90.47	335.99	100	516.28	334.32	101	425.81	−510.07	1361.69	0.371
Other healthcare costs	2242.73	133.20	100	1595.76	132.54	101	−646.98	−1018.00	−275.95	0.001
Mental health community/outpatient	1451.86	97.24	100	782.16	96.75	101	−669.70	−940.55	−398.86	<0.001
Non‐mental health outpatient	462.99	73.49	100	538.93	73.13	101	75.94	−128.77	280.65	0.465
Primary care	327.88	24.51	100	274.67	24.39	101	−53.21	−121.49	15.07	0.126
Social care	98.04	78.93	100	282.54	78.54	101	184.50	−35.36	404.35	0.100

	Placebo			Folic acid			Folic acid vs. placebo			
	Mean	SE	N	Mean	SE	N	Mean difference	95% LCL	95% UCL	*P* value
Total health and social care costs	6951.71	1247.91	94	5438.21	1268.41	91	−1513.50	−5032.79	2005.79	0.397
Total medication costs	1165.35	77.79	94	1117.09	79.07	91	−48.25	−267.63	171.12	0.665
Trial medication	980.02	69.65	94	1012.48	70.79	91	32.46	−163.96	228.89	0.745
Other medication	185.33	31.60	94	104.61	32.12	91	−80.72	−169.83	8.39	0.076
Total hospital costs	3620.69	1196.01	94	2106.62	1215.66	91	−1514.07	−4887.00	1858.86	0.377
Mental health inpatient	3521.14	1142.06	94	1537.02	1160.82	91	−1984.12	−5204.89	1236.64	0.226
Non‐mental health inpatient	99.55	360.91	94	569.60	366.84	91	470.05	−547.77	1487.87	0.363
Other healthcare costs	2032.00	141.38	94	1945.01	143.70	91	−86.99	−485.70	311.72	0.667
Mental health community/outpatient	1229.59	103.29	94	1092.71	104.99	91	−136.89	−428.18	154.41	0.355
Non‐mental health outpatient	517.69	78.74	94	532.08	80.03	91	14.39	−207.66	236.45	0.898
Primary care	284.72	25.51	94	320.22	25.93	91	35.50	−36.45	107.45	0.332
Social care	133.67	84.65	94	269.49	86.04	91	135.82	−102.90	374.53	0.263

Note

Excluding the participant who died during the trial.

N, number of participants; LCL, lower confidence level; SE, standard error; UCL, upper confidence level.

Folic acid comparison restricted to those participants who consented to separate randomization.

### 2.6 Role of the funding source

The CEQUEL study was funded by the Medical Research Council and managed by NIHR on behalf of the MRC‐NIHR partnership. Some study drug was donated by GlaxoSmithKline. Neither funder had any role in the study design; data collection, analysis or interpretation of data; writing of the report; or the decision to submit the paper for publication. The views expressed in this publication are those of the authors and not necessarily those of the MRC, NHS, NIHR or the Department of Health. The corresponding author had full access to all the data in the study and had final responsibility for the decision to submit for publication.

## 3 RESULTS

### 3.1 Outcomes

Both the available EQ‐5D utility values and the full imputed EQ‐5D dataset for baseline, 12 weeks and 52 weeks follow‐ups showed substantial improvement for all randomization groups during the 52 weeks follow‐up period (Table 5).

**Table 5:** Health‐related quality of life summary statistics for available cases and the full imputed dataset

EQ‐5D‐3L utility	N	Mean	SD	95% LCL	95% UCL
Lamotrigine vs placebo					
Available cases*					
Placebo					
Baseline	98	0.50	0.31	0.44	0.56
12 wk	76	0.63	0.29	0.56	0.70
52 wk	40	0.67	0.26	0.59	0.76
Lamotrigine					
Baseline	99	0.52	0.30	0.46	0.58
12 wk	76	0.66	0.32	0.59	0.74
52 wk	52	0.67	0.28	0.59	0.75
Imputed full dataset					
Placebo					
Baseline	100	0.50	0.31	0.44	0.56
12 wk	100	0.64	0.26	0.58	0.69
52 wk	100	0.66	0.19	0.63	0.70
Lamotrigine					
Baseline	101	0.52	0.30	0.46	0.58
12 wk	101	0.65	0.29	0.59	0.70
52 wk	101	0.66	0.23	0.61	0.70
Folic acid vs placebo^a^					
Available cases**					
Placebo					
Baseline	92	0.50	0.30	0.44	0.57
12 wk	72	0.65	0.29	0.59	0.72
52 wk	42	0.62	0.30	0.53	0.71
Folic acid					
Baseline	90	0.52	0.31	0.46	0.59
12 wk	68	0.66	0.32	0.59	0.74
52 wk	42	0.74	0.22	0.67	0.81
Imputed full dataset					
Placebo					
Baseline	94	0.51	0.30	0.45	0.57
12 wk	94	0.66	0.27	0.61	0.72
52 wk	94	0.64	0.23	0.59	0.69
Folic acid					
Baseline	91	0.52	0.31	0.46	0.59
12 wk	91	0.64	0.28	0.58	0.70
52 wk	91	0.69	0.20	0.65	0.73

Note

Excluding the participant who died during the trial.

LCL, lower confidence level; N, number of participants; SD, standard deviation; UCL, upper confidence level. Based on repeated measures mixed models of available cases, all randomization groups showed substantial improvement over 52 weeks (*P = 0.002, **P = 0.008), which was confirmed for the imputed data.

a Folic acid comparison restricted to those participants who consented to separate randomization.

Regression adjusted mean EQ‐5D utility and QALY differences between randomization groups are given in Table 3. No between‐group differences were seen for any of the group comparisons. Participants taking lamotrigine had slightly lower QALYs (−0.001; *P* = 0.972) than participants taking placebo, whereas participants randomized to folic acid had slightly higher QALYs (0.002, *P* = 0.938) than those allocated to placebo but these differences were neither statistically nor clinically significant. Relevant sensitivity analyses of the QALY calculation methods confirmed the main results and are therefore not presented separately.

### 3.2 Costs

Table 4 summarizes the regression adjusted mean costs per participant for the different resource use categories based on the full imputed dataset. Mean total costs from the health system perspective were £5,823 (SE: £1,156) for the lamotrigine group and £6,003 (SE: £1,162) for the placebo group and not significantly different overall (−£180, *P* = 0.913, n = 201). However, looking at subgroup categories, some potentially interesting differences emerged between the lamotrigine and placebo treatment groups. As expected, total medication costs were higher for the lamotrigine group (£713, *P* < 0.001), largely because of the cost of lamotrigine itself. However, other healthcare costs were significantly lower for the lamotrigine group (−£647, *P* = 0.001) driven by the overall lower mental health community/outpatient costs. Mental health and non‐mental health hospitalizations were the highest cost components, accounting for 42% and 48% of the total health and social care costs in the lamotrigine and placebo arms, respectively. Overall, inpatient costs were reported for 24 out of the 201 analysed participants (lamotrigine placebo: 14 participants; lamotrigine active: 10 participants) and were somewhat but not significantly higher in the placebo group (Table 4).

Folic acid was associated with lower mean total costs (n = 91, £5,438, SE: £1,268) than placebo (n = 94, £6,952, SE: £1,248) when controlled for lamotrigine, but the difference was not statistically significant (−£1,514, *P* = 0.397, n = 185; Table 4). The effect was dominated by the inpatient costs associated with one individual in the placebo group incurring total hospital costs of £97,670 during the 52 weeks follow‐up period. Numbers incurring inpatient costs were comparable (folic acid placebo: 11 participants; folic acid active: 13 participants). There were no statistically significant differences in any of the individual cost items.

Lost productivity due to work absence was analysed based on the available data. Of 119 participants, 41 were in employment at baseline and an additional six participants started working during the trial (n = 47). Of these 47 participants, 35 reported absenteeism due to (any) sickness during the follow‐up period for a total of 1,266 work days. Overall, the mean cost of lost productivity per patient in the placebo group significantly exceeded the lamotrigine group by £2,755 (*P* = 0.037), whereas no statistically significant difference was identified in the folic acid comparison.

Three outliers in terms of inpatient and overall health and social care costs were identified (two participants: active lamotrigine, active folic acid; one participant: placebo lamotrigine, placebo folic acid). Sensitivity analyses excluding these three participants confirmed the conclusions from the main analysis.

### 3.3 Cost‐effectiveness

**Figure 2:**
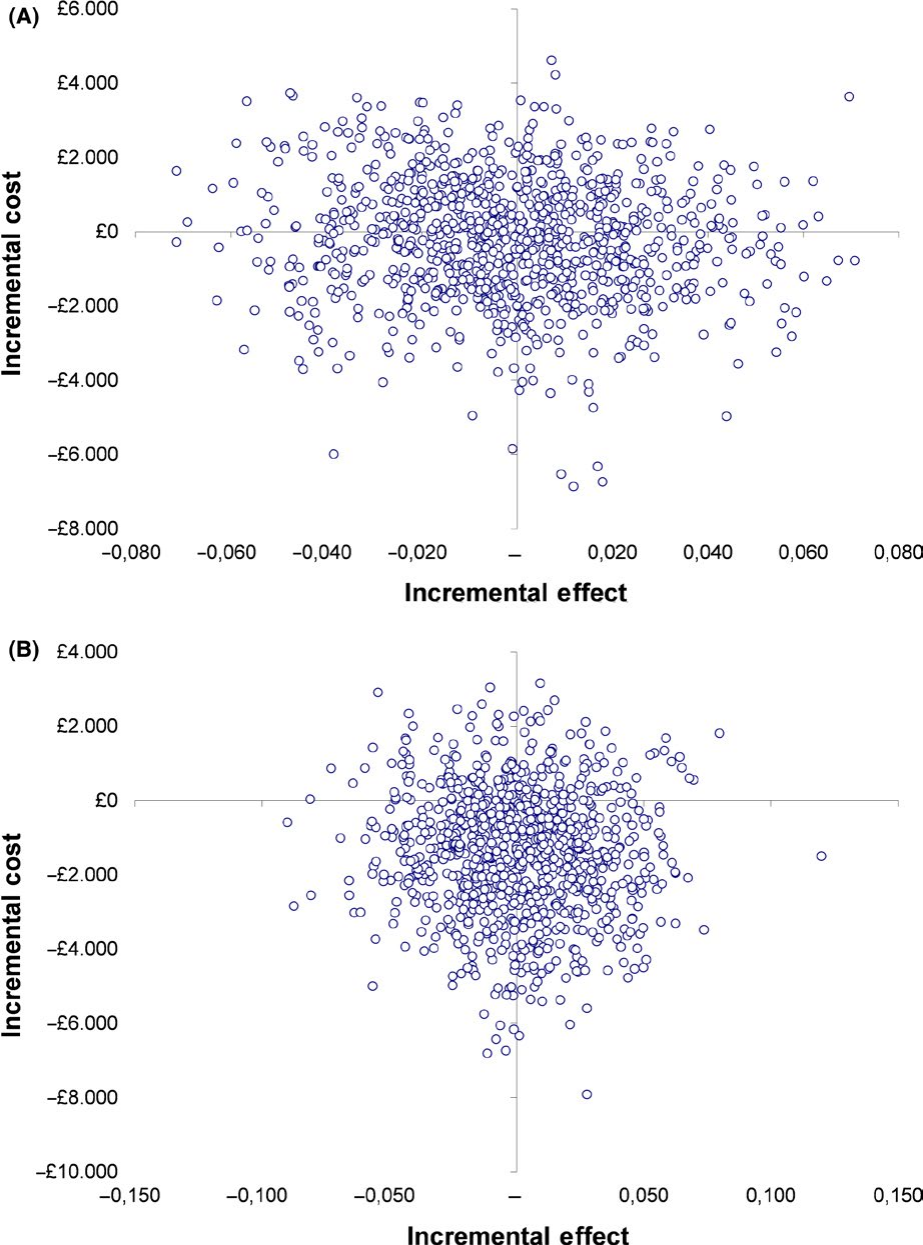
Bootstrapped mean differences in costs and effects by randomization: (A) health and social care costs, lamotrigine vs placebo, full imputed dataset (n = 201); (B) health and social care costs, folic acid vs placebo, full imputed dataset (n = 185) [Colour figure can be viewed at wileyonlinelibrary.com]

Figure 2 illustrates the scatterplots of the bootstrapped cost and effectiveness pairs for lamotrigine vs. placebo and folic acid vs placebo, respectively. The points in the scatter plot cover all four quadrants of the cost‐effectiveness plane, suggesting that there is significant uncertainty in the incremental cost‐effectiveness ratio. This is the result of the statistically non‐significant group differences between costs and outcomes (lamotrigine active vs lamotrigine placebo: incremental cost −£178, 95% LCI −3,394, 95% UCI 3,039, incremental effect −0.001, 95% LCI −0.050, 95% UCI 0.048; folic acid active vs folic acid placebo: incremental cost −£1,494, 95% LCI −4,755, 95% UCI 1,766, incremental effect 0.002, 95% LCI −0.051, 95% UCI 0.055).

## 4 DISCUSSION

In this economic evaluation of the effects of adding lamotrigine and folic acid to quetiapine therapy to treat bipolar depression, quality of life improved substantially for all groups during follow‐up with no significant differences in health outcomes between any of the group comparison. The same applies to costs, indicating no significant differences between groups in terms of total health and social care costs. Overall, this analysis suggests that the addition of lamotrigine to quetiapine is associated with good health outcomes and is cost neutral. Compared to placebo, folic acid produced neither clinical nor significant health economic benefits.

### 4.1 Quality of life

The effect of treatment of bipolar depression has so far not been systematically measured from a health economic perspective. The change from around 0.5‐0.7 for quality of life is both statistically and clinically significant. Regular mood monitoring may have contributed to this overall positive outcome as suggested by the earlier findings of Bopp and colleagues on steady improvement in patients using TrueColours.^34^ On the other hand, the EQ‐5D measure did not discriminate lamotrigine from placebo in the way that symptom ratings did. This is despite the fact that the EQ‐5D correlates quite well with depressive ratings on the QIDS scale in bipolar patients.^25^ Most likely, the EQ‐5D may also be confounded by an uncertain relationship to manic symptoms, because they may be viewed in a paradoxically positive way, whenever they occur. This makes it a potentially less reliable outcome measure for bipolar disorder than for major depression. This hypothesis on the responsiveness and reliability of the EQ‐5D in bipolar disorder needs further investigation.

### 4.2 Costs

Treatment costs were substantial, on average £6,000 per participant for the succeeding 52 weeks. This cost estimate is an underestimation of the real societal costs as it did not include estimates for the costs of unemployment, social benefits or patients’ out‐of‐pocket costs. Of the costs falling on the health and social care systems, hospitalization was, as expected, the largest, accounting for between 40% and 50% of total costs despite that only about 10% of the patients experienced hospitalization during the 52 weeks trial period (24 of 201 participants).

There was no difference in treatment costs between the lamotrigine or folate groups in comparison with their control groups. For the lamotrigine comparison, although trial medications costs were higher in the lamotrigine group than in the placebo group (£647, *P* < 0.001), mental health community/outpatient costs were lower (−£670, *P* < 0.001). The higher medication costs for lamotrigine and quetiapine combination therapy thus seem to be offset by savings in the mental health community/outpatient sector. This may reflect the improved clinical outcome implied by the self‐rated symptom data in the CEQUEL trial where lamotrigine treated patients did better than those treated with placebo. In the case of folic acid, by contrast, there were no apparent differences in any of the cost categories, just as there had been no difference in clinical outcomes.

In terms of total health and social care costs, estimated costs are at the lower end of the direct healthcare cost estimates derived in an earlier systematic literature review on the economic burden of bipolar disorders (US$ 8,000‐14,000 in 2009).^7^ Several model‐based economic evaluations on lamotrigine or quetiapine were identified in another systematic review.^34^ They are, however, not comparable to this cost‐utility analysis as none of them investigated the effect of lamotrigine and quetiapine combined, while healthcare system differences in the included studies may also contribute to these cost variations.^34^

Based on the results of this study, the lower mean productivity loss for the lamotrigine group suggests that quetiapine and lamotrigine combination therapy could result in further patient benefits and cost savings for the society. Given that bipolar disorders most commonly start in the early adulthood and the highest disease burden occurs in the working‐age population,^1^ this further emphasizes that the societal cost of lost productivity should be assessed in future economic evaluations.

## 5 LIMITATIONS

Firstly, due to difficulties in collecting information on informal care utilization using the TrueColours instrument, these indirect costs could not be incorporated in the sensitivity analysis from the broader societal perspective. Secondly, with full information over the 52 weeks follow‐up period for medication and inpatient use only, data on other health and social care use for 82 participants had to be fully and for 38 patients partially imputed. A sensitivity analysis based on available cases data (n = 119), however, supports the conclusions from the main analysis (Supporting Information Table S2). Furthermore, the reasons for drop out in the CEQUEL trial were the same in the different arms as were the numbers.^9^ Thirdly, the aim of the clinical study was to balance the group allocation for bipolar disorder type, age and sex (Table 1). In terms of health‐related quality of life, however, it seems noteworthy that some imbalances existed. For the group not allocated to the folic acid comparison, mean EQ‐5D utilities at baseline (0.427, SD = 0.315) were (statistically non‐significantly) lower than in all other groups. This suggests that opting out from the folic acid component of the study correlated with a lower quality of life. All outcome analyses were adjusted for EQ‐5D baselines utilities to adjust for any relevant differences. Fourthly, although total hospital costs were found to be non‐significantly lower in the lamotrigine group and in the folic acid group than in the placebo groups, these results are not robust due to the insufficient sample and event sizes for such inferences (inpatient resource use was only reported for 24 participants in total). Finally, the trial was not powered to detect interaction effects. A sensitivity analysis (Supporting Information Table S3) including an interaction dummy for both lamotrigine and folic acid combination therapy, however, suggests overall higher mean health and social care costs for participants taking quetiapine, lamotrigine and folic acid combined and thus supports the original clinical findings on the negative impact of concomitant lamotrigine and folic acid therapies. Finally, variables such as educational level, income, occupation, marital status, number of previous episode will also affect outcomes, but there is no reason to expect an impact on lamotrigine response per se.

## 6 CONCLUSIONS

This health economic analysis complements and amplifies the clinical findings^9^ and has important clinical and policy implications in terms of supporting calls for a wider use of lamotrigine to treat bipolar depression in published consensus guidelines.

## DISCLOSURES

JS, JRG, AG, JR and SM declare no competing interests. GMG is past president of ECNP, holds a grant from the Wellcome Trust, holds shares in P1vital and has served as consultant, advisor or CME speaker for Allergan, Angelini, Compass pathways, MSD, Lundbeck (/Otsuka or/Takeda), Medscape, Minervra, P1Vital, Pfizer, Servier, Shire, Sun Pharma.
