# Supplementary material for: Comparative economic evaluation of quetiapine plus lamotrigine combination vs quetiapine monotherapy (and folic acid vs placebo) in patients with bipolar depression (CEQUEL)
Source: Bipolar Disord. 2018 Dec 17;20(8):733–45. doi: 10.1111/bdi.12713 (PMC6491973; doi:10.1111/bdi.12713)
Supplement: Supplementary file 1 [file BDI-20-733-s001.docx]

### Supporting Information

**Table 1: Sensitivity analysis I: Imputed, regression adjusted mean annual costs per participant without outliers (in £ for the year 2013/14)**

|  | **Placebo** | | | **Lamotrigine** | | | **Lamotrigine vs. placebo** | | | |
| --- | --- | --- | --- | --- | --- | --- | --- | --- | --- | --- |
|  | **Mean** | **(SE)** | **N** | **Mean** | **(SE)** | **N** | **Mean difference** | **95% LCL** | **95% UCL** | **p value** |
| **Total health and social care costs** | 5101·98 | (777·36) | 99 | 4682·52 | (777·36) | 99 | -419·47 | -2590·70 | 1751·77 | 0·704 |
| **Total medication costs** | 802·12 | (77·19) | 99 | 1499·95 | (77·19) | 99 | 697·83 | 482·23 | 913·42 | <0·001 |
| Trial medication | 675·99 | (68·77) | 99 | 1312·69 | (68·77) | 99 | 636·70 | 444·61 | 828·79 | <0·001 |
| Other medication | 126·13 | (34·93) | 99 | 187·26 | (34·93) | 99 | 61·13 | -36·44 | 158·69 | 0·218 |
| **Total hospital costs** | 1978·25 | (716·87) | 99 | 1328·01 | (716·87) | 99 | -650·23 | -2652·53 | 1352·06 | 0·523 |
| Mental health inpatient | 1874·83 | (715·85) | 99 | 1288·20 | (715·85) | 99 | -586·63 | -2586·07 | 1412·82 | 0·563 |
| Non-mental health inpatient | 103·41 | (50·20) | 99 | 39·81 | (50·20) | 99 | -63·61 | -203·82 | 76·61 | 0·372 |
| **Other health care costs** | 2226·64 | (134·26) | 99 | 1573·08 | (134·26) | 99 | -653·56 | -1028·57 | -278·54 | 0·001 |
| Mental health community/outpatient | 1444·49 | (98·24) | 99 | 766·87 | (98·24) | 99 | -677·62 | -952·02 | -403·22 | <0·001 |
| Non-mental health outpatient | 454·47 | (73·55) | 99 | 531·23 | (73·55) | 99 | 76·76 | -128·68 | 282·19 | 0·462 |
| Primary care | 327·68 | (24·67) | 99 | 274·98 | (24·67) | 99 | -52·70 | -121·61 | 16·21 | 0·133 |
| **Social care** | 94·98 | (79·95) | 99 | 281·48 | (79·95) | 99 | 186·50 | -36·82 | 409·81 | 0·101 |
|  | **Placebo** | | | **Folic acid** | | | **Folic acid vs. placebo** | | | |
|  | **Mean** | **(SE)** | **N** | **Mean** | **(SE)** | **N** | **Mean difference** | **95% LCL** | **95% UCL** | **p value** |
| **Total health and social care costs** | 5836·19 | (835·27) | 93 | 4334·47 | 853·92 | 89 | -1501·72 | -3864·13 | 860·69 | 0·211 |
| **Total medication costs** | 1165·48 | (77·81) | 93 | 1101·97 | (79·55) | 89 | -63·51 | -283·58 | 156·56 | 0·570 |
| Trial medication | 983·69 | (70·02) | 93 | 1005·94 | (71·58) | 89 | 22·25 | -175·79 | 220·29 | 0·825 |
| Other medication | 181·79 | (31·51) | 93 | 96·03 | (32·21) | 89 | -85·76 | -174·88 | 3·36 | 0·059 |
| **Total hospital costs** | 2524·53 | (770·77) | 93 | 1039·75 | (787·97) | 89 | -1484·78 | -3664·75 | 695·19 | 0·181 |
| Mental health inpatient | 2443·53 | (769·66) | 93 | 965·08 | (786·84) | 89 | -1478·45 | -3655·29 | 698·38 | 0·182 |
| Non-mental health inpatient | 81·00 | (53·96) | 93 | 74·67 | (55·16) | 89 | -6·33 | -158·94 | 146·28 | 0·935 |
| **Other health care costs** | 2015·86 | (142·60) | 93 | 1924·23 | (145·78) | 89 | -91·63 | -494·94 | 311·67 | 0·654 |
| Mental health community/outpatient | 1223·45 | (104·43) | 93 | 1078·40 | (106·76) | 89 | -145·05 | -440·40 | 150·30 | 0·334 |
| Non-mental health outpatient | 507·63 | (78·85) | 93 | 525·01 | (80·61) | 89 | 17·38 | -205·64 | 240·39 | 0·878 |
| Primary care | 284·78 | (25·69) | 93 | 320·81 | (26·26) | 89 | 36·03 | -36·62 | 108·69 | 0·329 |
| **Social care** | 130·32 | (85·79) | 93 | 268·52 | (87·71) | 89 | 138·20 | -104·45 | 380·86 | 0·263 |

Note: Excluding the participant who died during the trial and three participants (outliers in terms of inpatient hospital costs). *Folic acid comparison restricted to those participants who consented to separate randomisation. SE=standard error. N=number of participants. LCL=lower confidence level. UCL=upper confidence level.

**Table 2: Sensitivity analysis II: Regression adjusted, mean annual costs per participant based on available cases (in £ for the year 2013/14)**

|  | **Placebo** | | | **Lamotrigine** | | | **Lamotrigine vs. placebo** | | | |
| --- | --- | --- | --- | --- | --- | --- | --- | --- | --- | --- |
|  | **Mean** | **(SE)** | **N** | **Mean** | **(SE)** | **N** | **Mean difference** | **95% LCL** | **95% UCL** | **p value** |
| **Total medication costs** | 798·94 | (77·12) | 100 | 1512·33 | (76·74) | 101 | 713·39 | 498·57 | 928·20 | <0·001 |
| Trial medication | 670·55 | (68·40) | 100 | 1317·53 | (68·06) | 101 | 646·98 | 456·45 | 837·52 | <0·001 |
| Other medication | 128·39 | (34·90) | 100 | 194·80 | (34·73) | 101 | 66·40 | -30·82 | 163·62 | 0·180 |
| **Total hospital costs** | 2863·48 | (1,113·15) | 100 | 2432·67 | (1,107·61) | 101 | -430·81 | -3531·41 | 2669·80 | 0·784 |
| Mental health inpatient | 2773·01 | (1,063·19) | 100 | 1916·39 | (1,057·90) | 101 | -856·62 | -3818·07 | 2104·84 | 0·569 |
| Non-mental health inpatient | 90·47 | (335·99) | 100 | 516·28 | (334·32) | 101 | 425·81 | -510·07 | 1361·69 | 0·371 |
| **Other health care costs** | 2382·85 | (248·42) | 56 | 1773·12 | (234·08) | 63 | -609·72 | -1289·15 | 69·70 | 0·078 |
| Mental health community/outpatient | 1496·28 | (202·61) | 56 | 919·94 | (190·91) | 63 | -576·34 | -1130·46 | -22·22 | 0·042 |
| Non-mental health outpatient | 476·29 | (128·28) | 56 | 550·26 | (120·88) | 63 | 73·97 | -276·88 | 424·83 | 0·677 |
| Primary care | 410·28 | (57·45) | 56 | 302·92 | (54·14) | 63 | -107·36 | -264·49 | 49·77 | 0·179 |
| **Social care** | 51·70 | (138·43) | 56 | 391·94 | (130·44) | 63 | 340·24 | -38·35 | 718·83 | 0·078 |
| **Lost productivity (sick leave)** | 5202·20 | (947·72) | 19 | 2447·29 | (764·78) | 28 | -2754·91 | -5338·05 | -171·76 | 0·037 |
|  | **Placebo** | | | **Folic acid** | | | **Folic acid vs. placebo** | | | |
|  | **Mean** | **(SE)** | **N** | **Mean** | **(SE)** | **N** | **Mean difference** | **95% LCL** | **95% UCL** | **p value** |
| **Total medication costs** | 1165·35 | (77·79) | 94 | 1117·09 | (79·07) | 91 | -48·25 | -267·63 | 171·12 | 0·665 |
| Trial medication | 980·02 | (69·65) | 94 | 1012·48 | (70·79) | 91 | 32·46 | -163·96 | 228·89 | 0·745 |
| Other medication | 185·33 | (31·60) | 94 | 104·61 | (32·12) | 91 | -80·72 | -169·83 | 8·39 | 0·076 |
| **Total hospital costs** | 3620·69 | (1,196·01) | 94 | 2106·62 | (1,215·66) | 91 | -1514·07 | -4887·00 | 1858·86 | 0·377 |
| Mental health inpatient | 3521·14 | (1,142·06) | 94 | 1537·02 | (1,160·82) | 91 | -1984·12 | -5204·89 | 1236·64 | 0·226 |
| Non-mental health inpatient | 99·55 | (360·91) | 94 | 569·60 | (366·84) | 91 | 470·05 | -547·77 | 1487·87 | 0·363 |
| **Other health care costs** | 2179·64 | (277·90) | 48 | 2160·45 | (246·21) | 61 | -19·19 | -759·29 | 720·91 | 0·959 |
| Mental health community/outpatient | 1309·07 | (227·10) | 48 | 1221·20 | (201·20) | 61 | -87·87 | -692·67 | 516·93 | 0·774 |
| Non-mental health outpatient | 518·63 | (144·32) | 48 | 574·31 | (127·86) | 61 | 55·68 | -328·68 | 440·03 | 0·774 |
| Primary care | 351·94 | (63·53) | 48 | 364·95 | (56·28) | 61 | 13·01 | -156·18 | 182·19 | 0·879 |
| **Social care** | 95·38 | (155·69) | 48 | 376·04 | (137·93) | 61 | 280·65 | -133·97 | 695·27 | 0·182 |
| **Lost productivity (sick leave)** | 3850·36 | (905·93) | 20 | 3747·68 | (821·67) | 24 | -102·68 | -2662·21 | 2456·85 | 0·936 |

Note: Excluding the participant who died during the trial. *Folic acid comparison restricted to those participants who consented to separate randomisation. SE=standard error. N=number of participants. LCL=lower confidence level. UCL=upper confidence level.

**Table 3: Sensitivity analysis III: Imputed, regression adjusted, mean annual costs per participant by randomization including their interaction (in £ for the year 2013/14) (n=185)**

|  |  | **Lamotrigine** | | | | **Folic acid** | | | | **Interaction** | | | |
| --- | --- | --- | --- | --- | --- | --- | --- | --- | --- | --- | --- | --- | --- |
|  | **N** | **Mean difference** | **95% LCL** | **95% UCL** | **p value** | **Mean difference** | **95% LCL** | **95% UCL** | **p value** | **Mean difference** | **95% LCL** | **95% UCL** | **p value** |
| **Total health and social care costs** | 185 | -3639·03 | -8514·98 | 1236·92 | 0·143 | -5012·70 | -9973·54 | -51·85 | 0·048 | 6917·35 | -48·32 | 13883·02 | 0·052 |
| **Total medication costs** | 185 | 762·34 | 455·16 | 1069·53 | <0·001 | -44·93 | -357·46 | 267·60 | 0·777 | -6·57 | -445·40 | 432·27 | 0·976 |
| Trial medication | 185 | 668·92 | 394·02 | 943·82 | <0·001 | -11·35 | -291·04 | 268·34 | 0·936 | 86·61 | -306·11 | 479·33 | 0·664 |
| Other medication | 185 | 93·42 | -30·99 | 217·83 | 0·140 | -33·58 | -160·16 | 92·99 | 0·601 | -93·18 | -270·90 | 84·55 | 0·302 |
| **Total hospital costs** | 185 | -3232·37 | -7920·05 | 1455·31 | 0·175 | -4340·32 | -9109·61 | 428·97 | 0·074 | 5587·04 | -1109·67 | 12283·74 | 0·101 |
| Mental health inpatient | 185 | -3113·48 | -7600·57 | 1373·61 | 0·173 | -4207·06 | -8772·27 | 358·15 | 0·071 | 4394·39 | -2015·76 | 10804·53 | 0·178 |
| Non-mental health inpatient | 185 | -118·89 | -1538·79 | 1301·01 | 0·869 | -133·26 | -1577·88 | 1311·36 | 0·856 | 1192·65 | -835·79 | 3221·09 | 0·247 |
| **Other health care costs** | 185 | -1105·76 | -1657·96 | -553·57 | <0·001 | -490·60 | -1052·41 | 71·21 | 0·087 | 797·86 | 9·01 | 1586·71 | 0·047 |
| Mental health community/outpatient | 185 | -967·54 | -1372·35 | -562·74 | <0·001 | -382·53 | -794·38 | 29·33 | 0·068 | 485·59 | -92·70 | 1063·88 | 0·099 |
| Non-mental health outpatient | 185 | -108·41 | -417·04 | 200·22 | 0·489 | -170·84 | -484·85 | 143·17 | 0·284 | 366·18 | -74·73 | 807·09 | 0·103 |
| Primary care | 185 | -29·81 | -130·40 | 70·79 | 0·559 | 62·77 | -39·58 | 165·12 | 0·228 | -53·91 | -197·62 | 89·80 | 0·460 |
| **Social care** | 185 | -63·24 | -392·84 | 266·36 | 0·705 | -136·85 | -472·19 | 198·49 | 0·422 | 539·02 | 68·16 | 1009·88 | 0·025 |

Note: Excluding the participant who died during the trial. Overall comparison restricted to those participants who also consented to folic acid randomisation. N=number of participants. LCL=lower confidence level. UCL=upper confidence level.
